# Emerging strategies in CAR-T cell therapy for acute myeloid leukemia: overcoming heterogeneity and improving safety through dual-antigen targeting

**DOI:** 10.1186/s40164-025-00726-4

**Published:** 2025-11-27

**Authors:** Ángeles Ocaña-Cara, Tuna Mutis, Jort J. van der Schans

**Affiliations:** Department of Hematology, Amsterdam UMC, Location VU University Medical Center, Cancer Center Amsterdam, De Boelelaan 1117, 1081HV Amsterdam, The Netherlands

**Keywords:** Acute myeloid leukemia, Chimeric antigen receptor T cell, Immunotherapy, Antigen expression, Dual-antigen targeting

## Abstract

While CAR-T cell therapy has been very successful for treating B cell malignancies, and more recently multiple myeloma, achieving clinical success for acute myeloid leukemia (AML) remains a significant challenge. The examination of current single-antigen targeting CAR-T cell studies for AML illustrates the challenges faced by this therapy: efficacy limitations arise from the heterogeneity of the disease, which often results in antigen escape and subsequent circumvention of single-antigen targeting CAR-T cells, while safety limitations are mainly due to undesired hematological toxicity stemming from the absence of an antigen specifically expressed on AML tumor cells and not on normal hematopoietic cells. This study offers a comprehensive analysis of the most relevant AML surface antigenic markers —CD123, CD33, ADGRE2, CLL-1, TIM-3, CD70, among others— along with their expression patterns across key cell types, including leukemic blasts, leukemic stem cells, hematopoietic stem cells and progenitors, adult blood cells, and other tissues. Additionally, a variety of strategies for developing CAR-T therapies with improved efficacy and specificity are explored, with dual-antigen targeting CAR-T cell therapies emerging as the most promising approach to overcome the major hurdles observed in single-antigen targeting CAR-T cell therapies. Overall, this review identifies dual-antigen targeting as a therapy holding great prospects in the search of an effective and safe therapeutic approach for AML patients.

## Introduction

Acute myeloid leukemia (AML) is a highly aggressive and heterogeneous hematological malignancy of the bone marrow that has an annual incidence of 4.3 per 100,000 in the United States, increasing with age [1]. Accounting for about 80% of cases, AML is the most frequent type of acute leukemia in adults [2]. AML is a clonal disorder characterized by excessive proliferation and expansion of a clonal population of hematopoietic stem cells (HSC) or other more differentiated progenitors of the myeloid lineage, leading to abnormal accumulation of leukemic blasts (LB) and disruption of normal hematopoietic differentiation [3]. This malignant expansion is initiated and maintained by leukemic stem cells (LSC), which have self-renewal capacity, similar to that of HSC [3]. Highly purified LSC fraction (CD34^+^ CD38^−^) from patient-derived AML xenografts has demonstrated the ability to entirely reconstitute AML tumor heterogeneity and disease in mouse models [4].

While conventional AML therapies such as chemotherapy and targeted therapies can achieve remission, they are not curative and are often followed by relapse due to residual LSC survival [5–7]. Allogenic hematopoietic stem cell transplantation (HSCT) is the oldest form of immunotherapy and a potentially curative option for AML patients through the graft versus leukemia (GvL) effect, particularly for those with high-risk disease determined by cytogenetic and molecular profiles, those relapsing after complete remission (CR) with chemotherapy or other treatments, and those who fail to respond to chemotherapy [8–10]. However, allogenic HSCT has several limitations, such as the need for a human leukocyte antigen (HLA)-matched donor, the possibility of failure in reconstituting a normal hematopoietic immune system, the risk of severe toxicities like graft versus host disease (GvHD) against the patient’s healthy tissues, and the risk of post-transplant relapse [11]. Unfortunately, over 30% of AML patients relapse after allogenic HSCT, and the 5-year overall survival rate remains between 30 and 50% [12–14]. The significant frequency of relapse and relatively poor survival outcomes showcase the need for improved immunotherapies for AML.

Prompted by the curative potential of immune cell-based treatments and their success in other hematological malignancies —such as chimeric antigen receptor (CAR)-T cell therapies for B cell hematologic malignancies [15, 16]—research has increasingly focused on CAR-T cell therapy for AML. However, progress has been limited, primarily due to challenges in identifying AML-specific targets. This difficulty results from two major obstacles: (1) a high overlap in antigen expression between leukemic cells (LB and LSC) and normal hematopoietic cells [17, 18], which can lead to severe hematotoxicities, thereby giving rise to safety concerns, and (2) inter- and intra-patient tumor cell heterogeneity, which results in antigen escape and efficacy limitations [17–20]. Together, these issues highlight the need for optimization of AML CAR-T therapy to enhance tumor-specific cytotoxicity and reduce target-related adverse effects.

Building on this background, this review aims to summarize the latest advancements in CAR-T cell therapy for AML and examine the challenges encountered, along with potential strategies to address them. To do so, we will review the current knowledge on the potential AML antigens for CAR targeting and their expression across leukemic and normal cell types. We will then compile the latest studies on single-antigen targeting CAR-T cell therapies, with accompanying success rates and major hurdles. Next, we will review the most recent approaches within the scientific community to improve efficacy and safety in CAR-T cell therapy. Finally, we will provide an overview of the latest novel CAR-T cell therapy designs involving dual-antigen targeting.

## AML antigen targets for CAR-T cell therapy

In 2017, Perna et al*.* established criteria for an ideal CAR target, whose main objective is to eradicate the tumor while minimizing intolerable damage to normal tissue [21]. Such a target should be expressed on all (or most) AML cells in most of the patients to address heterogeneity and, particularly, on LSC to prevent relapse. Preferentially, it should be co-expressed on LSC and LB to limit antigen escape, and present at high density to fully activate CAR-T cells. Additionally, the antigen should not be expressed on vital tissues or organs to avoid systemic toxicity, nor on normal hematopoietic stem progenitor cells (HSPC) to minimize on-target off-tumor hematotoxicities. Lastly, it should preferably be absent on T cells to prevent CAR-T cells from targeting themselves (i.e., fratricide), though this could be solved by knocking out the target antigen on the CAR-T cell surface [17, 21, 22]. To date, no AML tumor antigen fully complying with this ideal expression pattern has been identified. Nevertheless, several candidates are currently under investigation with the most prominent shown in Table 1. This review classifies them based on their presence or absence on CD34⁺ CD38⁻ cells, which include HSC and early hematopoietic progenitors.

**Table 1:** Expression of the main AML targets across different cell populations

Targets	Leukemic blasts	Leukemic stem cells (CD34^+^ CD38^−^)	Hematopoietic stem cells and early progenitors (CD34^+^ CD38^−^)	Hematopoietic late progenitors (CD34^+^ CD38^+^)	Hematopoietic adult cells	Non-hematopoietic organs	References
CD123 (IL-3Rα)	**Expressed** 80–98% **Antigen density** Medium MFI* *Detected in all (or nearly all) AML patients*	**Expressed** 80–96% **Antigen density** Medium MFI *Detected in all (or nearly all) AML patients*	**Expressed** 2.5–5% **Antigen density** Low MFI HSC: 726 molec/cell MPP: 617 molec/cell MLP: 1931 molec/cell	**Expressed** 9% (particularly in CMP and GMP) **Antigen density** Low MFI CP: 1709 molec/cell	**Expressed** Monocytes **Antigen density** Monocytes: low MFI, 1473 molec/cell B cells: negative MFI, 697 molec/cell Granulocytes: negative MFI, 478 molec/cell	Adipocytes and endothelial cells Gastrointestinal and respiratory tract epithelial tissues and other organs like bladder, brain, heart, kidney, liver, pancreas, lungs, muscles and reproductive structures	Tettamanti et al. (2013) [24] Pizzitola et al. (2014) [25] Ehninger et al. (2014) [30] Testa et al. (2014) [33] Perna et al. (2017) [21] Haubner et al. (2019) [28] Haubner et al. (2023) [29]
CD33 (Siglec-3)	**Expressed** 88–98% **Antigen density** High MFI *Detected in all (or nearly all) AML patients*	**Expressed** 77–90% Antigen density Medium MFI *Detected in all (or nearly all) AML patients*	**Expressed** 2.5–5% **Antigen density** Medium MFI HSC: 1951 molec/cell MPP: 1451 molec/cell MLP: 2455 molec/cell	**Expressed** 8% (particularly in myeloid progenitor cells) **Antigen density** Medium MFI CP: 3088 molec/cell	**Expressed** Monocytes and granulocytes **Antigen density** Monocytes: high MFI, 23,722 molec/cell Granulocytes: high MFI, 3912 molec/cell	Lung, skin, prostate	Taussig et al. (2005) [34] Ehninger et al. (2014) [30] Perna et al. (2017) [21] Haubner et al. (2019) [28] Haubner et al. (2023) [29]
ADGRE2 (EMR2/CD312)	**Expressed** 82–93% *Detected in all (or nearly all) AML patients*	**Expressed** 75–91% **Antigen density** 79% of AML patients display > 10^3^ ADGRE2 molecules/cell *Detected in 95% AML patients*	**Expressed** 2.5–5% **Antigen density** HSC: 880 molec/cell MPP: 772 molec/cell MLP: 1131 molec/cell	**Expressed** < 5% **Antigen density** CP: 668 molec/cell	**Expressed** Monocytes **Antigen density** Monocytes: 1569 molec/cell	Low or absent	Perna et al. (2017) [21] Lin et al. (2017) [35] Shahswar et al. (2018) [32] Haubner et al. (2023) [29] Huang et al. (2024) [31]
CLL-1 (CLEC12A/CD371)	**Expressed** 54–88% **Antigen density** Medium MFI *Detected only in some AML patients*	**Expressed** 14–77% **Antigen density** Negative MFI *Detected only in 60% of AML patients*	**Not expressed** < 2.5% **Antigen density** Negative MFI HSC:—molec/cell MPP:—molec/cell MLP:—molec/cell	**Expressed** 20% **Antigen density** Negative MFI CP: 810 molec/cell	**Expressed** Monocytes and granulocytes **Antigen density** Monocytes: high MFI, 11,812 molec/cell Granulocytes: high MFI, 6516 molec/cell	Low or absent	Perna et al. (2017) [21] Haubner et al. (2019) [28] Ma et al. (2019) [51] Haubner et al. (2023) [29]
TIM-3 (HAVCR2/CD366)	**Expressed** 80–87% **Antigen density** Low MFI *Detected in all (or nearly all) AML patients*	**Expressed** 65–79% **Antigen density** Low MFI *Detected in all (or nearly all) AML patients*	**Not expressed** NA **Antigen density** Negative MFI	**Expressed** Fraction of GMP committed to monocyte lineage **Antigen density** Negative MFI	**Expressed** Monocytes, fraction of NK cells and IFN-γ-producing CD4 + and CD8 + T cells **Antigen density** Monocytes: low MFI Granulocytes: negative MFI Lymphocytes: negative MFI	Bladder	Kikushige et al. (2010) [47] Jan et al. (2011) [52] Kikushige et al. (2013) [53] Haubner et al. (2019) [28]
CD70	**Expressed** 45–86% *Detected in some to most AML patients*	**Expressed** 30- > 75% *Detected in some to most AML patients*	**Not expressed** < 2.5%	**Not expressed** < 2.5%	**Expressed** Activated lymphocytes and monocytes	Low or absent	Nolte et al. (2009) [56] Perna et al. (2017) [21] Riether et al. (2017) [48] Shahswar et al. (2018) [32] Sauer et al. (2021) [49] Wu et al. (2023) [54]

^*^MFI (ratio) = MFI antigen-specific antibody / MFI isotype control. Categorized into negative (< 1.5), low (1.5–5), medium (5–15), or high (> 15)

HSC, hematopoietic stem cells; MFI, median fluorescence intensity; MPP, multipotent progenitor; MLP, multi-lymphoid progenitor; CP, common progenitor; CMP, common myeloid progenitor; GMP, granulocyte monocyte progenitor

### AML antigens present on HSC and early hematopoietic progenitors

These antigens are preferentially expressed on leukemic cells compared to their normal myeloid counterparts, meaning that they are highly expressed on the surface of LB and LSC, yet also present to some extent in normal CD34⁺ CD38⁻ HSC/early hematopoietic progenitors. The elevated expression in malignant cells relative to healthy cells may render them more susceptible to CAR-T cell therapy. The most studied examples are interleukin-3 receptor alpha chain (IL-3Rα/CD123) [23–25], sialic acid binding Ig-like lectin 3 (Siglec-3/CD33) [26] and adhesion G protein-coupled receptor E2/EGF-like module-containing mucin-like hormone receptor-like 2 (ADGRE2/EMR2/CD312) [27]. CD123 and CD33 are present on most LB (80–98%) and LSC (77–96%) at medium to high density [21, 28–30] (Table 1). ADGRE2 is also expressed on the majority of AML cells, albeit at a slightly lower frequency [21, 29, 31, 32] (Table 1). All three are additionally present at low to medium density in normal CD34⁺ CD38⁺ late hematopoietic progenitors (5–9%) and CD34⁺ CD38⁻ HSC/early hematopoietic progenitors (2.5–5%) [21, 28, 29, 33–35] (Table 1). They are also expressed on monocytes, and CD33 on granulocytes as well, which poses a risk of monocytopenia and granulocytopenia as a result of the therapy [29] (Table 1). Furthermore, although CD123 and CD33 are well-studied immunotherapy targets for AML, they are present in several compromising tissues/organs [21] (Table 1).

Less studied AML preferentially expressed antigens include CD7, expressed on approximately 30% of patients and associated with more aggressive disease and therapy resistance [36, 37]; however, its presence on T cells, natural killer (NK) cells, and a subset of the CD34^+^ CD38^−^ compartment associated with early lymphoid differentiation increases the risk of severe hematotoxicity when targeting this antigen [36, 38]. Likewise, Feline McDonough Sarcoma-like tyrosine kinase 3 (FLT3) is found on LB in a comparable percentage of patients with activating mutations [39, 40], but its presence on normal HSPC also raises safety concerns [40, 41]. The carbohydrate antigen Lewis Y tetrasaccharide (Le^Y^) further illustrates this issue, as it is often overexpressed in cancers like AML [17, 42–44] yet has a baseline expression on normal CD34^+^ myeloid progenitors and epithelial tissues [45]. Together, these antigens highlight the challenge of balancing therapeutic efficacy against on-target off-tumor effects in AML immunotherapy.

### AML antigens absent on HSC and early hematopoietic progenitors

Alternatively, CAR targets against AML could involve antigens expressed on LB and LSC, entirely absent on CD34^+^ CD38^−^ HSC/early progenitors, yet present on late hematopoietic progenitors and/or adult hematopoietic cells. This approach could be considered acceptable by relying on the capacity of HSC to differentiate into the entire spectrum of blood cells and to mitigate CAR-T cell induced on-target off-tumor hematotoxicity. On this basis, several membrane antigens are being investigated such as Human C-type lectin-like molecule-1/C-Type Lectin Domain Family 12 Member A (CLL-1/CLEC12A/CD371) [46], T-cell immunoglobulin and mucin domain 3 /Hepatitis A virus cellular receptor 2 (TIM-3/HAVCR2/CD366) [47] and CD70 [48, 49]. Collectively, these antigens could be referred to as selectively-expressed/HSC-sparing antigens.

Various studies have shown that CLL-1 is expressed on a considerable proportion of LB (71–88%) with moderate density [21, 28, 50, 51], although Haubner et al*.* reported only 54% [29] (Table 1). In contrast, CLL-1 expression on LSC appears more heterogeneous, showing variability across patients and studies, ranging from 77% [21] to 45% [50] and as low as 14% [29]. Additionally, CLL-1 is not present in all AML patients; Haubner et al. found CLL-1 expression on LSC in 45% at diagnosis and 20% at relapse [28], while their later study found detectable levels in 60% of patients [29]. These discrepancies highlight the variability in CLL-1 antigen coverage among AML populations. Similar to CD33, CLL-1 is also present on monocytes and granulocytes, thus potentially leading to comparable toxicities [29, 51], but no major expression was observed in non-myeloid tissues [21, 28] (Table 1).

Although their mean expression is lower than that of CD123 and CD33, TIM-3 and CD70 are other currently investigated HSC-sparing antigens present on the majority of AML cells (Table 1). More than 80% of bulk AML cells and over 65% of the LSC-enriched fraction express TIM-3 in most AML subtypes [21, 28, 47, 52, 53] (Table 1). However, its presence in healthy hematopoiesis, including activated T cells, and in the bladder, raises concerns for CAR targeting due to risks of fratricide and organ toxicity [28, 47] (Table 1). Conversely, CD70 expression has been found to be highly heterogeneous at multiple levels: the proportion of patients with detectable CD70 ranges from 96 to 100% [21, 48] to as low as 30% [32]; the frequency of CD70-positive cells varies widely (e.g., 45–86% in LB and 30–75% in LSC [21, 32, 48, 54]); and CD70 expression levels (i.e., antigen density) also differ between samples [49]. Notably, CD70 has been shown to be upregulated in LSC in response to treatment with hypomethylating agents —a standard of care for elderly or medically non-fit AML patients— [55], which may partly explain the discrepancies across studies. Despite this, CD70 is absent on normal HSPC and healthy tissues [21, 49, 54, 56], and in blood cells, it is expressed only on activated monocytes and lymphocytes, not under homeostatic conditions (Table 1) [48, 54, 56].

Cyclic ADP ribose hydrolase (CD38) is another CAR candidate since it is overexpressed on AML blasts and absent on CD34^+^ CD38^−^ HSC/early progenitors, as indicated by the surface markers used to classify this cell population [57]. In healthy humans, its expression pattern includes various immune cells, from progenitors to mature lineages [58]. A limitation of this glycoprotein is that, similar to HSC, it is not expressed on CD34^+^ CD38^−^ LSC, meaning that although targeting CD38 can eliminate most malignant blasts, it may not prevent AML relapse.

Additional HSC-sparing antigens —less extensively investigated and whose expression on AML cells remains to be fully characterized— include C–C Motif Chemokine Receptor 1 (CCR1) [21, 59], Leukocyte immunoglobulin-like receptor subfamily B member 2 (LILRB2) [21, 32], T cell activation increased late expression protein (Tactile/CD96) [60] and Tumor necrosis factor receptor superfamily member 1B (TNFRSF1B) [21]. Perna et al. report expression of all four on over 75% of AML cells [21], whereas Shahswar et al. observed lower frequencies for CCR1 and LILRB2, restricted to subsets of patients [32]. These antigens are also present on specific hematopoietic lineages (e.g., CCR1 on monocytes, T cells and NK cells [61]; LILRB2 on myeloid cells and B cells [21]; CD96 on T cells [21]; and TNFRSF1B on T cells and myeloid-rich organs [21]).

Lastly, Natural killer group 2D ligand (NKG2DL) is typically expressed on infected or malignant cells, such as those in various solid and hematological cancers like AML, but not on healthy cells [62, 63]. It binds to the NKG2D receptor on NK cells, activating their cytotoxic effector activity against infected/tumor cells [62]. However, as an escape mechanism, malignant cells often downregulate NKG2DL expression [62]. Therefore, despite being frequently present on the surface of AML cells, NKG2DL density is often low to evade immune clearance [17, 19].

## Single-antigen targeting CAR-T cell therapy for AML

To date, no CAR-T cell therapy has been approved for treating AML. There is significant effort ongoing to carry out preclinical studies targeting the antigens discussed in the previous section and to translate findings into clinical research. Several phase I and phase I/II clinical trials are currently recruiting patients. Although a few have been completed or terminated, many remain to be evaluated, leaving most results still pending. The majority of the current clinical trials focus on targeting CD123 (> 10 trials), CD33 (> 10 trials), and CLL-1 (> 5 trials), with additional trials targeting other antigens such as CD70, NKG2DL, CD7, FLT3, Lewis Y, and ADGRE2. Table 2 provides a summary of the reported findings on single-antigen targeting CAR-T cell therapies for AML.

**Table 2:** Clinical and preclinical trials of single-antigen targeting CAR-T cell therapies for AML

Target	Study type	Reference	Summary of results
CD123	Preclinical	Mardiros et al. (2013) [64] Gill et al. (2014) [65]	Efficacy: strong. High anti-leukemia activity in vitro (CD123 + cell lines and primary AML patient samples) and in vivo (mice) Safety: limited. No elimination of granulocyte/macrophage or erythroid lineages in vitro, but elimination of normal human myelopoiesis in vivo (mice). Reduced lysis of normal HSC achieved using CAR with light and heavy chains from different CD123-specific monoclonal antibodies
	Clinical	Budde et al. (2017) [66] Baghwat et al. (2024) [67]	Efficacy: controversial. Meaningful anti-leukemic responses including MRD-negative remissions in some patients (NCT02159495). Only 25% response due to cytokine-driven resistance mechanisms (NCT03766126) Safety: strong. Manageable and reversible toxicities (NCT02159495)
CD33	Preclinical	Marin et al. (2010) [71] Dutour et al. (2012) [70] Kenderian et al. (2015) [72]	Efficacy: strong. Sustained anti-AML activity in vitro and in vivo Safety: limited. Cytopenia and reduction in myeloid progenitors in vivo (xenograft models of hematopoietic toxicity)
	Clinical	Wang et al. (2015) [73] Tambaro et al. (2021) [74] Shah et al. (2023) [75]	Efficacy: limited. Decrease in AML blasts after two weeks, followed by relapse (NCT01864902). All patients died due to disease progression (NCT03126864). Rare MRD-negative remissions only at the highest dose level, occasionally followed by relapse (NCT03971799) Safety: controversial. Manageable adverse events (NCT03126864). Generally manageable, but significant toxicities associated with higher doses (NCT03971799)
ADGRE2	Preclinical	Unglaub et al. (2023) [82]	Efficacy: strong. Significant anti-leukemia activity in vitro after 48 h
	Clinical	NA	1 phase I clinical trial ongoing → no results available yet
CD7	Preclinical	Gomes-Silva et al. (2019) [36]	Efficacy: strong. Effective anti-leukemia effect in vitro (CD7 + AML cell lines and primary CD7 + AML) of CD7KO CD7 CAR T cells Safety: strong. No toxicity on myeloid or erythroid progenitor cells and progeny in vitro
	Clinical	Lu et al. (2024) [37]	Efficacy: limited. Complete remission in 7/10 patients; 3 underwent second allo-HSCT, with one remaining leukemia-free and two dying without relapse, while the other 4 relapsed within 90 days (NCT04938115) Safety: strong. Well-tolerated, 80% mild CRS and no neurotoxicity (NCT04938115)
FLT3	Preclinical	Chen et al. (2017) [83] Wang et al. (2018) [39] Niswander et al. (2023) [84]	Efficacy: strong. Significant anti-leukemia activity in vitro and in vivo (mouse models) Safety: limited. Not effect on HSC from human blood in vitro, but some reactivity against normal hematopoietic and non-hematopoietic tissues
	Clinical	NA	5 phase I/II clinical trials ongoing, 1 phase I withdrawn → no results available yet
Lewis Y	Preclinical	Peinert et al. (2010) [44]	Efficacy: strong. Specific killing of Le^Y^-positive AML target cells in vitro and in vivo in a myeloma mouse model Safety: -
	Clinical	Ritchie et al. (2013) [88]	Efficacy: limited. Transient reduction of blasts or remission in 2/4 patients, but relapse in all patients in 28 days-23 months (CTX 08–0002) Safety: strong. Grade 3 or 4 toxicity was not observed (CTX 08–0002)
CLL-1	Preclinical	Tashiro et al. (2017) [76] Wang et al. (2018) [77]	Efficacy: strong. High anti-leukemia activity in vitro (CLL-1 + AML cell lines and primary AML patient samples) and in vivo (human xenograft mouse model) Safety: strong. Normal HSC not targeted in vivo
	Clinical	Zhang et al. (2021) [78] Geyer et al. (2025) [79]	Efficacy: strong. Complete remission and no minimal residual disease in 3/4 patients (NCT03222674). Tumor clearance in 3/5 patients (NCT06017258) Safety: strong. Low grade/controllable adverse events (NCT03222674). No evidence of GvHD or dose-limiting toxicities at low dose (NCT06017258)
TIM-3	Preclinical	Lee et al. (2021) [87]	Efficacy: strong. Antileukemia activity in vitro (against AML cell lines and primary AML blasts) and in vivo (mouse models) Safety: -
	Clinical	NA	–
CD70	Preclinical	Sauer et al. (2021) [49] Leick et al. (2022) [81] Cheng et al. (2023) [80]	Efficacy: strong. Effective anti-tumor activity in vitro and in vivo. Combination with epigenetic modulators can enhance efficacy by upregulating CD70 expression Safety: strong. No HSC toxicity
	Clinical	NA	< 5 Phase I/II clinical trials ongoing → no results available yet
CD38	Preclinical	Yoshida et al. (2016) [85] Glisovic-Aplenc et al. (2023) [58]	Efficacy: strong. Potent anti-AML activity in vitro and in vivo; efficacy enhanced by all-trans retinoic acid Safety: limited. Expected in vivo reduction in hematopoietic progenitors
	Clinical	Cui et al. (2021) [92]	Efficacy: strong. Complete remission achieved in half of the patients (NCT04351022) Safety: strong. Mild and manageable adverse events (NCT04351022)
NKG2DL	Preclinical	Driouk et al. (2020) [86]	Efficacy: strong. High anti-leukemia activity in vitro, even at low NKG2DL expression, which can be selectively enhanced with HDAC inhibition Safety: strong. Healthy peripheral blood mononuclear cells (PBMC) not affected by NKG2DL-CAR-T cells + HDAC inhibition
	Clinical	Baumeister et al. (2019) [89] Sallman et al. (2019) [90]	Efficacy: controversial. No tumor responses at low doses, transient hematologic improvement in 1/6 patients at high doses (NCT02203825). Promising response rates of 46% (NCT03018405) Safety: strong. No dose-limiting toxicities or CAR-T cell-related adverse events (NCT02203825). No major adverse events (NCT03018405)

Preclinical CD123-CAR-T cell studies demonstrated strong anti-leukemia effects [64]; however, these were accompanied by loss of normal hematopoiesis in vivo due to CD123 expression on HSC [65], which may raise concerns about safety and potential hematotoxicities when translated into the clinic (Table 2). Despite this, multiple clinical trials with this strategy are taking place, although complete study results have not been made available yet. Early experience from a current phase I trial at the City of Hope (NCT02159495) involved 6 refractory AML patients post-HSCT and showed that CD123-CAR-T therapy can induce meaningful anti-leukemic responses, including substantial LB reduction in all cases, as well as morphologic leukemic-free states and sustained CR with undetectable minimal residual disease (MRD) in some, with manageable and reversible toxicities [66] (Table 2). However, more recently, a pilot report from a phase I trial of CD123-CAR-T cells in 12 relapsed/refractory AML patients (NCT03766126) showed that only 25% achieved a clinical response [67] (Table 2). The study found that the limited efficacy was due to a resistance pathway unique to AML: the therapy induced myeloid-supporting cytokines that maintained LB survival via kinase signaling, thereby promoting CAR-T cell exhaustion (Table 2) [67]. On the other hand, apart from their anti-leukemic effect, the ability of CD123-CAR-T cells to eliminate HSC can also be exploited as a bridging regimen for allogenic HSCT, as shown by Yao et al. [68].

CD33 seemed to be a highly promising target for CAR-T cells as the only drug currently approved for AML is the CD33 antibody–cytotoxic drug conjugate Gemtuzumab ozogamicin [69]. Preclinical studies showed potent anti-leukemic activity of CD33-CAR-T cells in vitro and in vivo [70–72]; however, this efficacy was limited in clinical studies, where LB reductions were generally temporary, leading to eventual disease progression in most patients (NCT01864902, NCT03126864) [73, 74], while MRD-negative remissions occurred rarely and only at the highest dose level, occasionally also followed by relapse (NCT03971799) [75] (Table 2). Importantly, the safety profile of the therapy remains controversial, with earlier, smaller studies suggesting manageable adverse events (NCT03126864) [74], while later experience indicated that the higher doses required for clinical efficacy were associated with significant toxicities (NCT03971799) [75] (Table 2).

In contrast, CLL-1 and CD70 CAR targeting seem to hold more potential, although further trials are needed to better prove their therapeutic value. CLL-1-CAR-T cells have shown strong preclinical results regarding efficacy and safety [76, 77], which appear to be consistent when translated into the clinic (NCT03222674) [78] (NCT06017258) [79] (Table 2). For CD70, although no clinical study results are available yet, its expression profile together with preclinical studies [49, 80, 81] (Table 2) may suggest a positive outcome in clinical trials.

Regarding other AML antigens, preclinical studies have demonstrated anti-leukemic activity for CAR-T cells against ADGRE2 [82], CD7 [36], FLT3 [83, 84], Lewis Y [44], CD38 [58, 85], and NKG2DL [86] (Table 2). However, their clinical translation remains limited or uncertain. FLT3 safety profile in vitro [39] (Table 2), and ADGRE2 expression on HSC (Table 1), suggest potential toxicities that may limit clinical success. TIM-3-CAR-T cells show preclinical efficacy in vivo [87] but lack safety assessments or clinical data (Table 2). The Le^Y^-CAR-T trial (CTX 08–0002), the first to demonstrate CAR-T cell activity in AML, showed limited efficacy, with all patients relapsing [88] (Table 2). Similarly, CD7-CAR-T trial (NCT04938115) also demonstrated modest responses with frequent relapses [37] (Table 2). NKG2DL-CAR-T cells show variable efficacy in clinical trials (NCT02203825, NCT03018405) [89, 90] (Table 2), possibly due to epigenetic and post-translational regulation [91]. Lastly, CD38-CAR-T cells demonstrated promising activity, with half of patients achieving CR and manageable toxicities in a small clinical study (NCT04351022) [92] (Table 2); however, further studies are needed to confirm efficacy and safety.

Overall, when translated into the clinical setting, single-antigen targeting CAR-T cell therapy for AML has shown considerable limitations in efficacy and safety. Additionally, it may not be sufficient to eliminate all LB and LSC due to heterogeneous antigen expression, leading to relapses. Altogether, these drawbacks showcase the need for the design of improved CAR-T cell strategies for AML.

## Improving efficacy and safety of CAR-T cell therapies for AML

### Strategies to improve therapeutic efficacy

Tumor escape through antigen loss is a key factor constraining CAR-T cell efficacy. LB and LSC present intrinsic immune evasion strategies, such as mutations that impair immune response, downregulation of HLA molecules that undermines antigen presentation, and alterations in cytokine expression [93]. To counteract these mechanisms, CAR-T cell efficacy can be enhanced by increasing antigen expression on tumor cells. For example, histone deacetylase (HDAC) inhibition upregulates NKG2DL, which is otherwise expressed at low levels, improving the cytotoxicity of NKG2DL-CAR-T cells [86]; epigenetic modulators have been shown to increase CD70 expression on LSC in a clinical trial (NCT03030612) [55], and to effectively enhance the anti-tumor activity of CD70-CAR-T cells in a preclinical study [80]; and all-trans retinoic acid can boost CD38 expression to improve CD38-targeting efficacy [85].

The tumor microenvironment (TME) also plays a central role in limiting persistence and anti-leukemic activity of CAR-T cells [93]. In addition to the cell-intrinsic resistance mentioned above, the bone marrow niche further contributes to the suppression of anti-tumor immunity through the accumulation of metabolic byproducts (e.g., lactate, adenosine, and kynurenine), the expansion of regulatory T cells, myeloid-derived suppressor cells (MDSC) and M2 macrophages, and vascular remodeling that promotes hypoxia and hampers immune cell migration [93]. Collectively, these factors create a hostile milieu that impairs CAR-T cell function; therefore, TME modulation is increasingly recognized as a critical strategy to enhance CAR-T cell activity, which can be approached in multiple ways [94].

An approach for TME modulation focuses on overcoming immune checkpoint inhibition of CAR-T cells. Programmed cell death 1 (PD-1) expression is induced on the surface of CAR-T cells when they are antigen-stimulated for a sustained period of time [95]. PD-1 can bind to its ligand (PD-L1) —a potent immunosuppressive molecule found on AML blasts— and inhibit T cell activation and cytotoxic effector activity, leading to exhaustion [96]. Approaches to mitigate this include combining CAR-T cells with exogenous checkpoint blockade with antibodies (e.g., anti-PD-1 or anti-CTLA-4), genetic engineering of CAR-T cells to secrete these antibodies, or direct knock-out or downregulation of inhibitory receptors such as PD-1 within the CAR-T product [17]. For example, Lin et al. introduced a PD-1 silencing short hairpin RNA (shRNA) sequence into CLL-1-CAR-T cells and found that PD-1 knock-down enhanced CLL-1-CAR-T cells’ ability to eliminate AML tumor cells in vitro [97].

Cytokine-based strategies also enhance CAR-T efficacy. “Armored” CAR-T cells are engineered to produce proinflammatory cytokines in addition to the expression of a CAR to enhance cytotoxicity and alleviate immune suppression [98]. Often, the production of the cytokine is triggered by CAR signaling to localize cytokine secretion to the tumor microenvironment, a design known as fourth-generation CAR-T cells or T-cell Redirected for Universal Cytokine Killing (TRUCK) [99, 100]. The power of “armored” approaches was exemplified in a recent report of the CLEAR-AML phase I trial (NCT06017258) [79]. The study evaluated IL-18-secreting CLL-1-CAR-T cells in 5 refractory AML patients and demonstrated enhanced cytotoxicity and antitumor activity that resulted in tumor clearance (i.e., MRD negativity) in 3 patients, with manageable toxicities [79]. In addition, based on the previously discussed evidence that AML can exploit therapy-induced myeloid-supporting cytokines, including Granulocyte–Macrophage Colony-Stimulating Factor (GM-CSF), FLT3L and IL-3, to sustain blast survival and promote CAR-T cell exhaustion [67], combination with cytokine signaling inhibitors is a rational strategy to further improve efficacy. Alternatively, combination with drugs that eliminate the MDSC population [101], inhibitors of metabolic enzymes to reduce TME metabolites [102], or immunomodulators such as TGF-β inhibitors [103] also aim to create a more permissive TME for CAR-T cell function.

Beyond TME modulation strategies, other efficacy-enhancing approaches focus on improving CAR-T cell sensitivity. For example, Mansilla-Soto et al. developed an HLA-independent T cell receptor (HIT) and validated its high antigen sensitivity both in vitro and in vivo, compared to the most sensitive CAR design [104]. This could be interesting for lowly expressed AML antigens like CLL-1, CD70 and NKG2DL. Another option is to optimize the binding properties between CAR-T cells and tumor cells by testing different single-chain variable fragments (scFv) with varying affinities and evaluating the avidity (i.e., overall strength of CAR-antigen interactions across multiple binding sites) [100, 105]. Recent work has shown that CAR-T cells with intermediate avidity achieved the greatest anti-tumor activity, highlighting the importance of evaluating this parameter [106].

Further strategies aim at extending CAR-T cell persistence, enabling sustained anti-leukemic activity over time. Optimal selection of T cell subsets is a key determinant; while conventional CAR-T cells are generally derived from αβ T cells, γδ T cells have recently become a very attractive alternative because they combine multi-mechanistic killing through γδ T cell receptor (TCR)- and NK receptor-mediated pathways, provide rapid innate-like cytotoxicity for early anti-leukemic activity, exhibit durable persistence and memory in humans, and recognize targets in an HLA-independent manner, reducing GvHD risk and allowing “off-the-shelf” products [107, 108]. Allogenic Delta One T cells expressing a CD123-CAR (Vδ1 γδ T cells) showed potent in vitro and in vivo killing of AML blasts and maintained activity upon tumor rechallenge [109], while allogenic γδ CD33-CAR-T cells from healthy donors demonstrated rapid cytotoxicity, prolonged metabolic fitness, and superior in vivo anti-AML activity compared with conventional αβ CAR-T cells [110]. T cell differentiation state also affects persistence. Less differentiated T cell populations like naïve (T_N_), stem cell memory (T_SCM_), and central memory T cells (T_CM_), which primarily rely on oxidative phosphorylation and reduced glycolysis, offer prolonged survival and antitumor activity in vivo compared to effector memory (T_EM_) or terminal effector T cells (T_TE_) [111]. To favor these phenotypes, CAR-T cells can be preconditioned *ex vivo* with cytokines like IL-7, IL-15, or IL-21 [112, 113], exposed to epigenetic modulators [114], or be metabolically reprogrammed to reinforce these pathways [115, 116]. In AML, CD44v6-CAR-T cells have been shown to achieve enhanced memory and activation phenotypes, increased persistence, and boosted cytotoxicity when combined *ex vivo* with demethylating agents such as decitabine or azacitidine [117]. Finally, optimizing the CAR domain design can also enhance the in vivo durability of the product [118]. The incorporation of additional or alternative costimulatory domains (third-generation CARs) [100, 118, 119], along with cytokine expression-inducing domains (fourth-generation CARs/TRUCKs, as discussed above) [99, 100, 118], or IL-2 receptor STAT3/5 signaling domains that simulate cytokine recognition (fifth-generation CARs) [100, 118, 120], has also been shown to improve persistence of various CAR-T cells.

### Strategies to improve therapeutic safety

The expression of target antigens on healthy cells may result in on-target off-tumor toxicities, as observed in several studies described above. Furthermore, excessive immune activation mediated by CAR-T cells may result in Cytokine Release Syndrome (CRS) and Immune effector Cell-Associated Neurotoxicity Syndrome (ICANS), which are not necessarily related to the target antigen [17, 18]. These toxicities can be mitigated by controlling the persistence of CAR-T cells, enabling their selective inactivation when necessary. To this end, inducible suicide-based safety switches have been developed and shown to be effective in clinical settings for non-AML hematological malignancies [121, 122]. In AML research, Tashiro et al. developed and preclinically evaluated CLL-1-CAR-T cells carrying an inducible caspase-9 (iCASP9) suicide system, whose activity can be reduced upon administration of a specific drug to limit toxicities against adult myeloid cells [76]. Preliminary results from a clinical trial of CLL-1-CAR-T cells with an iCASP9 safety switch demonstrated high efficacy by achieving CR in 3 of 4 pediatric patients, and high safety since all adverse events were low-grade and manageable [78]. However, these promising safety results may also reflect the choice of a more specific target, such as CLL-1, rather than the switch itself. In line with this, Warda et al. plan to conduct a phase I clinical trial based on their CD123-CAR-T cell with iCASP switch preclinical studies that prompt controlled CAR-T cell ablation and suggest potential safety improvements for the therapy [123].

Antibody-dependent cellular cytotoxicity (ADCC) can also be used to deliberately eliminate CAR-T cells engineered to express antigens such as epidermal growth factor receptor (EGFR), CD20, or CD52 through the monoclonal antibodies Cetuximab, Rituximab, or Alemtuzumab, respectively [100]. For instance, preclinical antibody-mediated depletion of CD123-CAR-T cells at an optimal timepoint allowed leukemic cell clearance while preserving remission [124]. In non-AML hematological malignancies, CAR-T products containing antibody-based safety switches have been evaluated in clinical trials, including JCAR017 (EGFR/Cetuximab in non-Hodgkin lymphoma, NCT02631044) [125], and UCART19 (RQR8/Rituximab in B-cell acute lymphoblastic leukemia, NCT02746952) [126], reporting manageable CRS and neurotoxicity. However, it remains unclear whether switch-off systems fully mitigate risk, as therapy is often stopped only after hematotoxicity or other adverse effects are evident. Although both iCASP9 and antibody-based switch-off systems have shown generally favorable safety profiles in clinical trials, in most studies these outcomes reflect the overall CAR-T product rather than documented activation of the switch, leaving their specific rescue potential uncertain. Whether these strategies can rapidly reverse severe toxicities in AML remains to be addressed in future trials.

Whereas the strategies mentioned above can switch-off CAR-T cells, other approaches rely on switching them on. One example is universal CAR-T cell therapy, where activation only occurs in the presence of soluble scFvs fused to an element that binds to the corresponding module on membrane-bound CARs [127, 128]. Besides the possibility to switch to a different target antigen, this also allows activity to be controlled by administration or withdrawal of the soluble component. The adapter modules can have many different natures [128]. The effectiveness and temporal precision of the therapy can be influenced by the binding affinity, biodistribution, and size of the soluble scFvs, with larger molecules creating longer extracellular domains that may reduce T cell activation [129], and smaller molecules requiring repeated dosing to maintain anti-tumor responses due to rapid clearance [128], reflecting practical considerations in the design. Despite this, universal CD123-CAR-T cells are under evaluation in an ongoing phase I trial (NCT04230265) [130].

Messenger RNA (mRNA) electroporation represents another alternative for limiting in vivo active time of CAR-T cells, in which CAR genes remain episomal with transient expression [131]. However, a phase I trial using T cells electroporated with anti-CD123 CAR mRNA in AML patients yielded unsatisfactory results (NCT02623582) [132]. No treatment-related deaths or hematotoxicities were observed, but fever and varying degrees of CRS occurred in all patients [132]. Furthermore, the trial was terminated because the therapy failed to induce CD123-expressing leukemic cell death [132]. Therefore, RNA electroporation has so far proven not to be an appropriate method due to its lack of efficacy.

Other effector cell types have an intrinsically different safety profile compared to T cells and can be used as alternative platforms for CAR-based strategies. NK cells are inherently cytotoxic to virus-infected and malignant cells, enabling both antigen-independent killing through recognition of stressed or HLA-I–deficient cells, and antigen-dependent killing via ADCC [94, 133]. Noteworthy, CAR-NK cell therapy avoids GvHD and other CAR-T-related side effects like CRS or neurotoxicity [94, 133]. However, their short half-life, poor persistence, and resistance to viral transduction remain major limitations [133]. Cytokine-Induced Killer (CIK) cells are an in vitro-generated population with a mixed T (CD3^+^) and NK (CD56⁺) cell phenotype, derived from autologous/allogenic peripheral blood mononuclear cells (PBMC) through cytokine stimulation [134]. Because their killing is mainly driven by NK-like pathways through activating NK receptors, CIK cells mediate HLA-independent cytotoxicity and display very low alloreactivity, resulting in minimal GvHD even in haploidentical settings, unlike traditional αβ CAR-T cells [135]. These effector cell platforms extend beyond the scope of this review but have been discussed in detail elsewhere in the context of AML (NK [136–138]; CIK [139–142]).

## Dual-antigen targeting CAR-T cell therapy for AML

**Fig. 1:**
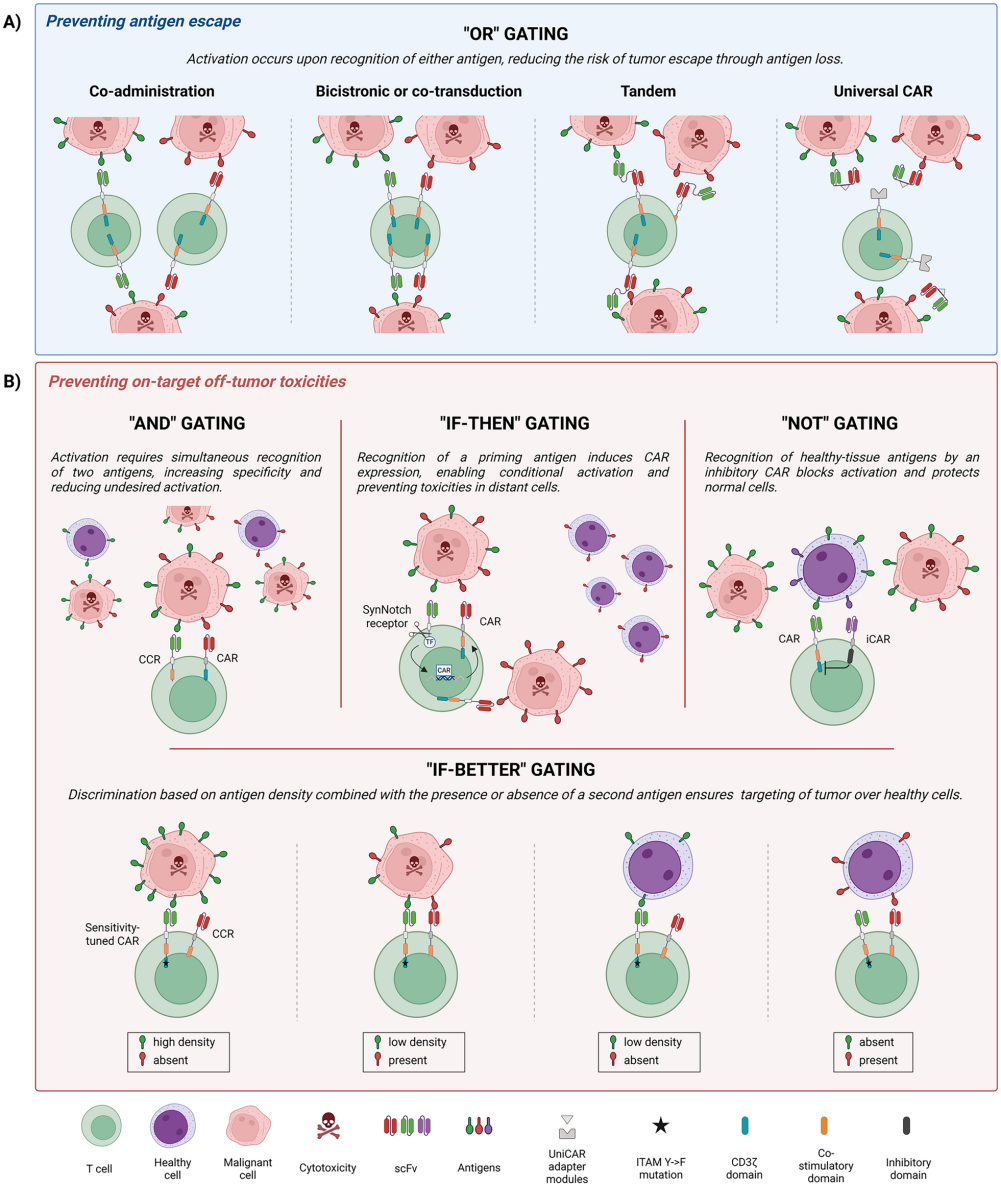
Boolean logic gating strategies for dual-antigen targeting CAR-T cell therapy in AML. **A** Strategies to enhance efficacy and prevent tumor antigen escape include “OR” gating, in which T cells exert cytotoxicity against any cell expressing at least one of two antigens. This can be achieved through co-administration of two CAR-T products with different targets, expression of two CARs in a single T cell, a single CAR containing two scFvs, or universal CAR-T cell platforms. **B** Strategies to enhance therapy safety and limit on-target off-tumor toxicities include: “AND” gating, which splits activation across a CAR and a CCR, triggering full T cell cytotoxicity only when both antigens are present; “IF–THEN” gating, which uses SynNotch receptor signaling to induce CAR expression, restricting killing to specific target sites; “NOT” gating, which blocks CAR-mediated activation upon recognition of a healthy-tissue antigen via an iCAR; and “IF-BETTER” gating, which enables selective cytotoxicity based on antigen density through a threshold-tuned CAR and presence of a second antigen via a CCR that enhances activation even if the threshold is not reached. Created in https://www.BioRender.com

Dual-antigen targeting may offer a solution for challenges encountered in single-antigen targeting CAR-T cell therapy for AML, such as tumor heterogeneity, antigen escape, and on-target off-tumor toxicities. It represents a breakthrough offering the potential to enhance both efficacy and safety. A wide range of dual-antigen targeting CAR options exist to promote the T cell cytotoxic effector functions using different Boolean logic gating strategies (Fig. 1). Table 3 shows various preclinical and clinical dual-antigen targeting CAR-T cell studies in AML.

**Table 3:** Dual-antigen targeting CAR-T cell strategies for AML

Gating	Strategy	Antigen targets	Study type	Reference
OR	Co-administration	NA	NA	NA
	Bicistronic/compound or Co-transduction	CD123, CD33 Bicistronic	Preclinical	Petrov et al. (2018) [145]
		CD123/CD33, CLL-1 Co-transduction	Preclinical	Atilla et al. (2022) [147]
		FLT3, NKG2DL Bicistronic	Preclinical	Li et al. (2022) [149]
		CD123/CLL-1, CD70 Co-transduction	Preclinical	Scherer et al. (2022) [148]
		CD123, CLL-1 Bicistronic	Preclinical	Xie et al. (2023) [150]
		CD123, NKG2DL Bicistronic	Preclinical	Jin et al. (2023) [151]
		CD123, CD33 Bicistronic	Preclinical	Ma et al. (2024) [146]
		CD33, CLL-1 Bicistronic	Phase I clinical trial	Liu et al. (2018) NCT05016063 [152]
		CD123, CD33 Bicistronic	Phase I clinical trial	Limongello et al. (2021) NCT04156256 [154]
	Tandem/bivalent	CD123, Folate Receptor ß	Preclinical	Ghamari et al. (2021) [155]
		CD33, CD146	Preclinical	Alberti et al. (2023) [141]
		CD123, CLL-1	Preclinical	Wang et al. (2024) [156]
		CD33, CLL-1	Preclinical	Wang et al. (2024) [157]
	UniCARs	CD123, CD33	Preclinical	Cartellieri et al. (2016) [127]
	Unknown strategy	CD33, CLL-1	Phase I clinical trial	NCT05248685 [17]
		CLL-1, CD33	Phase I clinical trial	NCT05467254. Not yet recruiting
		CD123, CLL-1	Phase II/III clinical trial	NCT03631576 [17]
		single CAR-T or double CAR-T cells with CD33, CD38, CD56, CD123, CD117, CD133, CD34 or Mucl	NA	NCT03473457 [17]. Terminated, the therapeutic effect was not expected
AND	Split signaling (CAR + CCR)	CD13, TIM-3	Preclinical	He et al. (2020) [163]
		CD123, CD33	Preclinical	Boucher et al. (2023) [164]
		CD33, TIM-3	Preclinical	Wang et al. (2023) [162]
		CLL-1, TIM-3	Preclinical	Van Der Schans et al. (2023) [161]
		NKG2DL, PD-L1	Preclinical	Sun et al. (2023) [165]
		CD123, CD33	Preclinical	Perriello et al. (2023) [142]
		WT1, CD33	Preclinical	Dao et al. (2024) [166]
	Unknown	CD123, TIM-3	Phase I/II clinical trial	NCT06125652
IF -THEN	SynNotch circuit	CD33, CD123	Preclinical	Jambon et al. (2025) [167]
NOT	CAR + iCAR	CD123/CD33/CD38, not CD93	Preclinical	Richards et al. (2021) [170]
		CLL-1, not CD15/CD16	Preclinical	Zhang et al. (2024) [171]
		CD33/CD43, not CD16b/CLEC9A	Preclinical	DiAndreth et al. (2025) [172]
IF -BETTER	Split signaling (sensitivity-tuned CAR + CCR)	ADGRE2, CLL-1	Preclinical + Phase I clinical trial	Haubner et al. (2023) [29] NCT05748197

### “OR” gating

The most widely explored logic strategy is the “OR” gating or “CAR-CAR” strategy that aims at minimizing the risk of antigen escape by enabling T cells to attack any cell expressing at least one of the two target antigens [28] (Fig. 1). This is particularly useful when the antigen levels are low or downregulated [100]. However, for it to reduce on-target off-tumor toxicities, neither antigen should be present in normal tissues, although low to moderate expression may be acceptable in certain cases [21].

Numerous attempts have sought the most optimal combination of AML antigen targets for “OR” gating, although many of these remain largely unexplored. Perna et al. performed a high-throughput proteomic and transcriptomic analysis to generate an AML surface protein dataset, and evaluated potential target pairs, identifying four combinations (CD33/ADGRE2; CLL-1/CCR1; CD33/CD70; and CLL-1/LILRB2) for future use in preclinical models [21]. Haubner et al. also generated an AML surface protein dataset by combining proteomic and flow cytometry data and identified CLL-1/TIM-3 as the most favorable pair for “CAR + CAR” approaches, enhancing efficacy without entailing greater toxicities [28]. Meanwhile, noting differences between children and adult AML surface protein expression, Willier et al. identified CD33/CLL-1 as the preferred combination for pediatric AML, although they cautioned that this approach may require a safety switch or subsequent HSCT [143].

Several methods exist to generate “OR” gating CAR-T products, which have been thoroughly studied in B cell malignancies and multiple myeloma and are now gaining interest in AML [144]. The simplest method is the coadministration (sequential or simultaneous) of two single-antigen targeting CAR-T cell therapies [144] (Fig. 1). For obtaining T cells co-expressing two CARs with different specificities, two methods can be employed: simultaneous co-transduction of two CAR vectors, producing a heterogeneous population expressing either or both CARs, or bicistronic/compound CAR-T products, where a single vector carries both CAR constructs [144] (Fig. 1). In 2018, Petrov et al. developed one of the first bicistronic AML CAR-T cell therapies targeting CD123 and CD33, showing cytotoxicity against CD123 + CD33- and CD123-CD33 + cell lines and increased survival in four leukemia mouse models [145] (Table 3). Accordingly, Ma et al. recently confirmed high anti-tumor activity of bicistronic CD123/CD33-CAR-T cell in vitro and in vivo [146] (Table 3). Studies using co-transduction of CARs that showed meaningful in vitro and in vivo control of AML tumor growth were developed by Atilla et al. targeting CLL-1 in combination with either CD123 or CD33 [147] and by Scherer et al. targeting CD70 with either CD123 or CLL-1 [148] (Table 3). Other bicistronic products with promising outcomes for reducing antigen escape target FLT3/NKG2DL [149], CD123/CLL-1 [150], and CD123/NKG2DL [151] (Table 3). Importantly, supported by promising preclinical data, Liu et al. conducted the first phase I clinical trial of a dual-antigen targeting CAR-T cell for AML patients (NCT05016063) [152] (Table 3). Bicistronic CD33/CLL-1-CAR-T cells were given to two AML patients, both achieving MRD-negative CR after two infusions (one of them after only 19 days), proving anti-tumor potency and effectiveness [152, 153]. However, this therapy led to pancytopenia, which therefore required allogenic HSCT, suggesting that it is not a safe standalone therapy [152, 153]. This adverse effect is not unexpected considering the expression profile of both antigens in the normal hematopoietic system (Table 1). Other ongoing dual-antigen “OR” gating trials are listed in Table 3 [17, 154].

Besides co-expression of CARs, “OR” gating can also be achieved with tandem or bivalent CARs, where two antigen-binding sites (i.e., scFv), each with a different specificity, share the same transmembrane and signaling domains [144] (Fig. 1). Preclinical studies show efficient elimination of AML cell lines and primary AML cells and confirmed efficacy with in vivo studies [141, 155–157] (Table 3). Universal CARs can also implement the “OR” logic, as in Cartellieri et al., where a dual-specific anti-CD123-CD33 targeting module, consisting of two scFv connected to a target epitope, interacts with the scFv of a universal CAR, increasing AML cell killing compared to simultaneous administration of single soluble modules [127] (Fig. 1, Table 3).

It is worth mentioning an innovative design by Teppert et al. using T cells co-transduced with a CD33 CAR and a mutant nucleophosmin 1 TCR (dNPM1-TCR) that specifically recognizes the AML neo-epitope CLAVEEVSL [158]. These cells demonstrated robust and long-lasting anti-tumor cytotoxicity in vitro, particularly against low-level antigen targets, and complete eradication and prevention of AML tumor growth in vivo [158]. Although it does not involve two CARs, this novel avenue could also be classified as an “OR” gating dual-antigen strategy.

### “AND” gating

A distinct logic strategy incorporated into the AML field is the “AND” gating [159]. Unlike “OR” gating, where CAR-T cells activate upon recognition of either antigen, “AND” gated CAR-T cells require simultaneous engagement of two targets, thereby increasing specificity and reducing undesired activation. This design splits signaling domains into two separate receptors: a CAR with the activating domain (CD3ζ) and a chimeric costimulatory receptor (CCR or cCAR) containing a costimulatory domain (CD28 and/or 4-1BB), hence full T cell activation only occurs when both receptors are engaged [159]. The goal is to spare single-positive cells and eliminate double-positive cells by selecting an antigen pair that is co-expressed on cancer cells, but not on healthy cells, thus allowing minor overlap of one antigen on healthy cells [28] (Fig. 1). However, careful balancing of CAR and CCR is required to prevent ‘leakiness’ of the “AND” gating strategy (i.e., elimination of single-positive cells for the CAR target). We and others have demonstrated that this balancing may be achieved by selecting a low affinity scFv for the CAR [159, 160], or by reducing signaling strength of the CAR [161].

Haubner et al. suggested CD33 and TIM-3 as an adequate pair for “AND” gating because, although CD33 has a broad expression profile, TIM-3 expression is more restricted, so overlap occurs mainly in AML tumor cells and possibly monocytes, sparing HSPC and vital tissues [28] (Table 1). Wang et al. compared CD33/TIM-3 dual-targeting bicistronic, tandem and split-signaling designs concluding that, while maintaining the efficacy, split-signaling offered the safest profile in vitro, with in vivo confirmation still pending [162] (Table 3). Another combination suggested by Haubner et al. for “AND” gating is CLL-1 and TIM-3, which is being evaluated in our group [161] (Table 3). Our results show that, upon simultaneous recognition of the two antigens, dual split-signaling TIM-3/CLL-1-CAR-T cells potently inhibit LB and LSC viability in vitro and prevent AML engraftment in vivo [161]. TIM-3 is also involved in two other “AND” gating studies: a preclinical study targeting TIM-3 and CD13 —aminopeptidase N, which is preferentially expressed LB— [163], and a phase I/II clinical study (NCT06125652) targeting TIM-3 and CD123 (Table 3). Information on the specific cell design in this trial is lacking, but the ClinicalTrials.gov entry suggests an “AND” strategy to improve LSC specificity and reduce normal HSPC killing. Additional combinations such as CD123/CD33 and NKG2DL/PDL-1 are being explored in preclinical studies [142, 164–166] (Table 3). For pediatric AML, Willier et al. proposed CD33/CLL-1 as the best antigen pairing for “CAR + CCR” approaches, although further in vivo and clinical studies are needed to determine whether targeting these two antigens provides sufficient coverage for AML eradication [143].

When comparing the two most studied logic gating strategies in AML, the “OR” gating stands out as an effective approach to limit antigen escape and enhance efficacy [28]. However, it may be preferentially regarded as a conditioning regimen before allogenic HSCT due to the higher safety risk associated with targeting cells expressing either of two antigens, which increases the likelihood of on-target off-tumor hematological toxicities [162]. Conversely, the “AND” gating could potentially provide a transplant-independent approach [162]. Its success, however, depends on the antigen selection; the two antigens should be broadly co-expressed across LSC to maintain efficacy, although antigen loss may still occur and pose a limitation [21]. Acknowledging this, the “AND” gating can combine strong anti-tumor efficacy and increased specificity for AML tumor cells, thereby lowering potential adverse effects [28].

### Other logic gating

A 2025 study by Jambon et al. introduced a nuanced strategy called the “IF-THEN” gating approach [167] (Table 3). In this design, recognition of a first antigen by a Synthetic Notch (SynNotch) receptor triggers a circuit that induces expression of a CAR on the T cell surface (Fig. 1). Consequently, CAR-mediated cytotoxicity occurs only after engagement with the first antigen, thereby confining CAR activation to specific target sites. This strategy represents a conditional, stepwise “AND” gating. Applied to dual targeting of CD33 and CD123, this system demonstrated high AML-killing efficacy both in vitro using AML cell lines and in vivo in AML patient-derived xenograft models [167]. Additionally, Jambon et al. claim that the CAR signal modulated by the SynNotch circuit is safe, as it spares HSPC, and helps ease CRS [167]. However, loss of either the SynNotch- or CAR-targeted antigen could compromise the therapy effectiveness. Furthermore, unintended killing of single-antigen tumor cells may still occur during the decay phase of SynNotch-gated CAR expression, as observed by others using a similar strategy [168].

The “NOT” logic gating, originally implemented by Fedorov et al. in 2013, makes use of an inhibitory CAR (iCAR) [169]. Here, T cells are engineered to express a conventional CAR targeting a tumor antigen and an iCAR that recognizes an antigen on healthy tissues, generating an inhibitory signal that blocks T cell activation upon engagement to prevent damage to normal cells [169] (Fig. 1). Several AML studies have applied this strategy, with CARs targeting antigens like CD123, CD33, and CLL-1, and iCARs inhibiting T cell activity when binding to endothelial antigens like CD93 [170], or myeloid lineage antigens like CD15 and CD16 [171], or CD16b and CLEC9A [172] (Table 3). Nevertheless, similar to single-antigen targeting, “AND”, and “IF–THEN” strategies, tumor antigen loss remains a challenge for this approach.

Another innovative logic strategy devised by Haubner et al. is the “IF-BETTER” gating approach, which enables selective cytotoxicity based on the density of antigen 1 and the presence or absence of antigen 2 [29] (Table 3). Particularly, they engineered a T cell, denoted as ADCLEC.syn1, that co-expresses a sensitivity-tuned CAR capable of recognizing ADGRE2 above a certain density threshold and a CCR that, upon CLL-1 recognition, enhances T cell activation even when the ADGRE2 density threshold is not surpassed (Fig. 1). In this way, they were able to specifically target LSC, which have moderate to high ADGRE2 expression and presence of CLL-1, and spare normal HSPC that have low ADGRE2 expression and lack CLL-1. This innovative approach thus achieved enhanced efficacy by overcoming antigen escape through dual-antigen targeting and enhanced safety by limiting undesired toxicity against normal HSPC. ADCLELC.syn1 is currently being evaluated in a phase I clinical trial (NCT05748197).

In conclusion, the numerous possibilities of targeting two different antigens illustrate the versatility and potential of dual-antigen targeting CAR-T cell therapies. While some gating strategies, such as the “OR” gating, have been extensively studied in preclinical settings, others like “AND”, “IF-THEN”, and “IF-BETTER” are more recent and innovative. Dual-antigen targeting offers a promising avenue for AML therapy that still requires further validation in clinical trials, but it also has some caveats. Limited CAR-T cell durability remains a challenge [173], although combining dual-targeting with persistence-enhancing strategies described earlier may be particularly beneficial. Moreover, the increased technical complexity of these designs requires careful optimization and can make manufacturing more challenging and costly. From the engineering standpoint, dual-antigen targeting often involves larger gene constructs, which can affect viral packaging capacity, or the use of multiple viral vectors, which can place additional stress on T cells, both of which can reduce transduction efficiency and complicate production [174]. Given the baseline transduction difficulties and the limited CAR integration and expression when the leukapheresis product contains mostly poorly proliferative cells [175], selecting cell subsets with higher proliferative capacity may be an additional manufacturing step, along with its associated challenges. Tonic signaling, or antigen-independent CAR activation from spontaneous scFv clustering, is another concern [176]. Its intensity depends on CAR design, stability, and the presence of positively charged patches within the scFv, making different CARs carry varying risk [177]. In dual-targeting CAR-T therapy, the risk of tonic signaling and subsequent T cell exhaustion may be amplified, particularly in tandem “OR”-gated CARs, where the proximity of the two scFvs can promote cross-clustering [178]. Lastly, the optimization process can also be more complex, as dual-antigen systems require coordinated fine-tuning of both receptors. Nevertheless, despite these limitations, it is expected that novel cutting-edge gating strategies enhancing AML CAR-T cell efficacy and safety will emerge as research advances.

## Conclusion

CAR-T cell therapy has emerged as a promising curative opportunity for AML, offering a potential alternative to chemotherapy and allogenic HSCT [6, 7]. Despite the success in other hematological malignancies [15, 16], AML presents unique challenges, mainly due to the lack of AML-specific antigens and disease heterogeneity [18, 19]. Single-antigen targeting CAR-T cell studies have generally failed to achieve effective tumor clearance without severe hematological toxicity.

To overcome these limitations, dual-antigen targeting CAR-T cell therapies have been explored in preclinical studies, employing diverse designs and showing potential to enhance anti-leukemic activity and curtail on-target off-tumor toxicities. Future research will focus on optimizing these strategies, including refinement of “AND”, “NOT”, and “IF-BETTER” gating circuits, to further improve CAR-T cell discrimination between malignant and healthy cells. Additionally, other Boolean logic approaches remain to be applied to AML [179]. Novel advanced dual-targeting strategies may also emerge, further improving efficacy and safety of the therapy. Overall, dual-antigen targeting CAR-T cells represent a leading avenue for AML treatment, offering a roadmap for future innovation and clinical translation.

## Data Availability

No datasets were generated or analysed during the current study.

## Abbreviations

ADCC: Antibody-dependent cellular cytotoxicity
ADGRE2: Adhesion G Protein-Coupled Receptor E2
AML: Acute myeloid leukemia
CAR: Chimeric antigen receptor
CCR: Chimeric costimulatory receptor
CCR1: C–C Motif Chemokine Receptor 1
CD: Cluster of differentiation
CIK: Cytokine-induced killer
CLEC12A: C-Type Lectin Domain Family 12 Member A
CLL-1: C-type lectin-like molecule-1
CMP: Common myeloid progenitor
CP: Common progenitor
CR: Complete remission
CRS: Cytokine release syndrome
EGFR: Epidermal growth factor receptor
EMR2: Epithelial growth factor-like module-containing mucin-like hormone receptor-like 2
FLT3: Feline McDonough Sarcoma-like tyrosine kinase 3
GMP: Granulocyte-monocyte progenitor
GvHD: Graft versus host disease
GvL: Graft versus leukemia
HAVCR2: Hepatitis A virus cellular receptor 2
HDAC: Histone deacetylase
HIT: HLA-independent TCR
HLA: Human leukocyte antigen
HSC: Hematopoietic stem cell
HSCT: Hematopoietic stem cell transplantation
HSPC: Hematopoietic stem progenitor cell
ICANS: Immune effector Cell-Associated Neurotoxicity Syndrome
iCAR: Inhibitory CAR
iCASP9: Inducible caspase-9
IFN-γ: Interferon gamma
IL-3Rα: Interleukin-3 receptor alpha chain
LB: Leukemic blast
Le^Y^: Lewis Y
LILRB2: Leukocyte immunoglobulin-like receptor subfamily B member 2
LSC: Leukemic stem cell
MDSC: Myeloid-derived suppressor cell
MFI: Median fluorescence intensity
MLP: Multi-lymphoid progenitor
MPP: Multipotent progenitor
MRD: Minimal residual disease
mRNA: Messenger RNA
NK: Natural Killer
NKG2DL: Natural killer group 2D ligand
NPM1: Nucleophosmin 1
PD-1: Programmed cell death 1
PD-L1: Programmed cell death 1 ligand
RNA: Ribonucleic acid
scFv: Single-chain variable fragment
shRNA: Short hairpin RNA
Siglec-3: Sialic acid binding Ig-like lectin 3
SynNotch: Synthetic Notch
T_CM_: Central memory T cell
T_EM_: Effector memory T cell
T_N_: Naïve T cell
T_SCM_: Stem cell memory T cell
T_TE_: Terminal effector T cell
TCR: T cell receptor
TIM-3: T-cell immunoglobulin and mucin domain 3
TME: Tumor microenvironment
TNFRSF1B: Tumor necrosis factor receptor superfamily member 1B
TRUCK: T-cell Redirected for Universal Cytokine Killing
WT1: Wilms tumor 1
